# 

**Published:** 1896-01

**Authors:** 


					﻿THE
Southern Medical Record.
A Monthly Journal of Medicine and Surgery.
Vol. XXVI. ATLANTA, GA., JANUARY, 1896.	No. 1?
ir	.	. r-
BERNARD WOLFF, M. D., Editor.
LOUIS H. JONES, M. D., Business Manager. ’
Associate Editors :
2. ,T. GRIGGS, M. D.	WILLIS F. WESTMORELAND, M. D.
j. McFaddengaston,m. d. | /	3 r\
Entered at the Post Office at Atlanta, Ga., as Second-Class Matter.
_ The Foote & Davies Co., Atlanta, Ga.
				

